# Interleukin-6 in Schizophrenia—Is There a Therapeutic Relevance?

**DOI:** 10.3389/fpsyt.2017.00221

**Published:** 2017-11-06

**Authors:** Milica Milovan Borovcanin, Ivan Jovanovic, Gordana Radosavljevic, Jelena Pantic, Slavica Minic Janicijevic, Nebojsa Arsenijevic, Miodrag L. Lukic

**Affiliations:** ^1^Department of Psychiatry, Faculty of Medical Sciences, University of Kragujevac, Kragujevac, Serbia; ^2^Center for Molecular Medicine and Stem Cell Research, Faculty of Medical Sciences, University of Kragujevac, Kragujevac, Serbia; ^3^Faculty of Medical Sciences, University of Kragujevac, Kragujevac, Serbia

**Keywords:** interleukin-6, schizophrenia, immune response, inflammation, metabolic syndrome

## Abstract

Renewing interest in immune aspects of schizophrenia and new findings about the brain-fat axis encourage us to discuss the possible role of interleukin-6 (IL-6) in schizophrenia. Previously, it was suggested that a primary alteration of the innate immune system may be relevant in schizophrenia. Functional dichotomy of IL-6 suggests that this chemical messenger may be responsible for regulating the balance between pro- and anti-inflammatory responses, with tissue-specific properties at the periphery and in the central nervous system. Specific phase of this chronic and deteriorating disorder must be considered, which can involve IL-6 in acute or possible chronic inflammation and/or autoimmunity. We give an overview of IL-6 role in the onset and progression of this disorder, also considering cognitive impairment and metabolic changes in patients with schizophrenia. Data suggest that decreased serum level of IL-6 following antipsychotic therapy could be predisposing factor for the development of obesity and obesity-related metabolic disorders in schizophrenia. As we reviewed, the IL-6 plays significant role in disease genesis and progression, so the use of specific inhibitors may not only be beneficial for exacerbation and alleviation of positive symptoms, but may attenuate cognitive impairment in patients with schizophrenia.

## Introduction

The immune system could be described as a sensory system whose primary purpose is identifying the foreign (“non-self”) substances, referred to as antigens. Two equally important aspects of the immune system are the innate and acquired immunity. The mechanisms of innate immunity are physical and chemical barriers, cellular components, and soluble molecules. The principal cellular components of the innate immune response include dendritic cells, monocytes, macrophages, granulocytes, and natural killer (NK) cells. The unique components of acquired immunity are T and B lymphocytes that specifically recognize and respond to an antigen. Thus, innate and acquired immune response represents the action of various specialized cells and soluble molecules that they secrete.

Cytokines are chemical messengers or hormones of the immune system. They mediate cell–cell interactions in immune responses and induce the movement of cells toward sites of inflammation, infection, and trauma. Thus, these soluble molecules regulate and coordinate many activities of the cells of innate and acquired immunity.

There is a renewing interest in immune aspects of schizophrenia (1, 2) and new findings have been presented regarding the linkage of innate and adaptive immunity by the brain-fat axis (3). These findings encouraged us to discuss a possible influence of interleukin-6 (IL-6) in schizophrenia onset and progression, considering cognitive impairment and metabolic changes in patients with schizophrenia. We try to enlighten some metabolic aspects of IL-6 in schizophrenia and introduce some new drug-targets.

## IL-6 as a Pleiotropic Cytokine

Interleukin-6 was first identified as a B-cell differentiation factor, which induces antibody production by activated B cells. This cytokine promotes the differentiation of B cells, the population expansion and activation of T cells, and regulates the acute inflammation (4, 5). Upon IL-6 binding to IL-6 receptor (IL-6R) are initiated its multiple functions. The IL-6R is composed of the IL-6-binding chain, existing in forms of transmembrane IL-6R and soluble IL-6R (sIL-6R) (6), and a gp130 signal-transducing chain (7). IL-6 is secreted by different types of cells and under various conditions of immune activation. For example, the primary sources of this cytokine are monocytes and macrophages at site of injury during acute inflammation, as well as T cells in chronic inflammation.

The toll-like receptors (TLRs) are major sensors of the innate immunity, able to recognize a broad spectrum molecule of different classes of microbes, as well as damage-associated molecular pattern released from stressed cells, and to initiate an inflammatory response rapidly. TLR ligation is one of the earliest events leading to IL-6 production (8). In homeostatic conditions, level of IL-6 is low, but IL-6 serum levels rise quickly in stress. Numerous studies show that IL-6 modulates various aspects of the innate immune system, such as hematopoiesis and influx of neutrophils at sites of infection or trauma (9, 10). In addition, this cytokine induces synthesis of C-reactive protein, serum amyloid A, and fibrinogen, as proteins of acute phase.

Interestingly, IL-6 has pro- and anti-inflammatory properties which are context dependent. Although it has been mostly regarded as a clear pro-inflammatory cytokine of acute innate responses, it has many regenerative or anti-inflammatory activities crucial for resolution of inflammation [reviewed in Ref. (11)]. A role of IL-6 in limiting inflammation has been based on several observations. It was shown that IL-6 exerts its immunosuppressive properties by inhibiting activity of the transcription factor named nuclear factor kappa-light-chain-enhancer of activated B cells and expression of the chemokine receptor on dendritic cells required for recruiting these cells to lymphoid tissues (12). Moreover, IL-6 signaling promotes alternative macrophages activation and inhibits their microbicidal activities (13–15). Additionally, IL-6 also induces expression of the IL-1R antagonist and the soluble p55 receptor for tumor-necrosis factor (TNF) (16). In these settings, IL-6 is involved not only in the induction of acute inflammation, but also in the resolution of inflammation.

Upon activation by antigen-presenting cells, naive CD4+ T cells can differentiate into Th1, Th2, or Th17 and regulatory T (Treg) cells. Besides its role in the innate immune response, IL-6 also regulates acquired immunity by promoting specific differentiation of naive CD4+ T cells, but these effects are context dependent. Some reports suggested that IL-6 skewed T-cell differentiation toward Th2 cells and simultaneously inhibited Th1 polarization through two independent molecular mechanisms (17, 18). However, it has been demonstrated that IL-6 promotes Th1-cell responses (19). Recently, it has been reported that IL-6 has an important role in regulating Th17/Treg balance (20). Thus, in the presence of the transforming growth factor-beta (TGF-β), IL-6 is a necessary signal for differentiation of naive T cells to Th17 cells, a subset of T helper cells that are implicated in the induction of autoimmune diseases (21, 22), and contribute to local tissue damage in chronic inflammatory diseases (23). In contrast, IL-6 can strongly inhibit the TGF-β-induced differentiation of Treg cells that inhibit autoimmunity and protect against tissue injury (24). Downregulation or overproduction of IL-6 alters the balance between Th17 and Treg cells. Th17/Treg disbalance appears to interfere with immunological tolerance and consequently leading to development of autoimmune and chronic inflammatory diseases (20). Also, considering its role in production of IL-10 by T cells (25, 26), it seems that IL-6 may be included in relieving an inflammatory response. This functional dichotomy suggests that IL-6 may be responsible for regulating the balance between pro- and anti-inflammatory responses.

## Role of IL-6 in the Brain Function

Interleukin-6 can be also produced by activated astrocytes and microglial cells in the brain (27, 28) and neurons (29, 30). IL-6Rs have been localized in the central nervous system (31). It has been shown that IL-6 boosts central neurotrophin secretion by different cells (32, 33). Under stress, IL-6 induces increased production of metabolites by astrocytes, while neurons primarily consume resources from the microenvironment (34).

**Figure 1:**
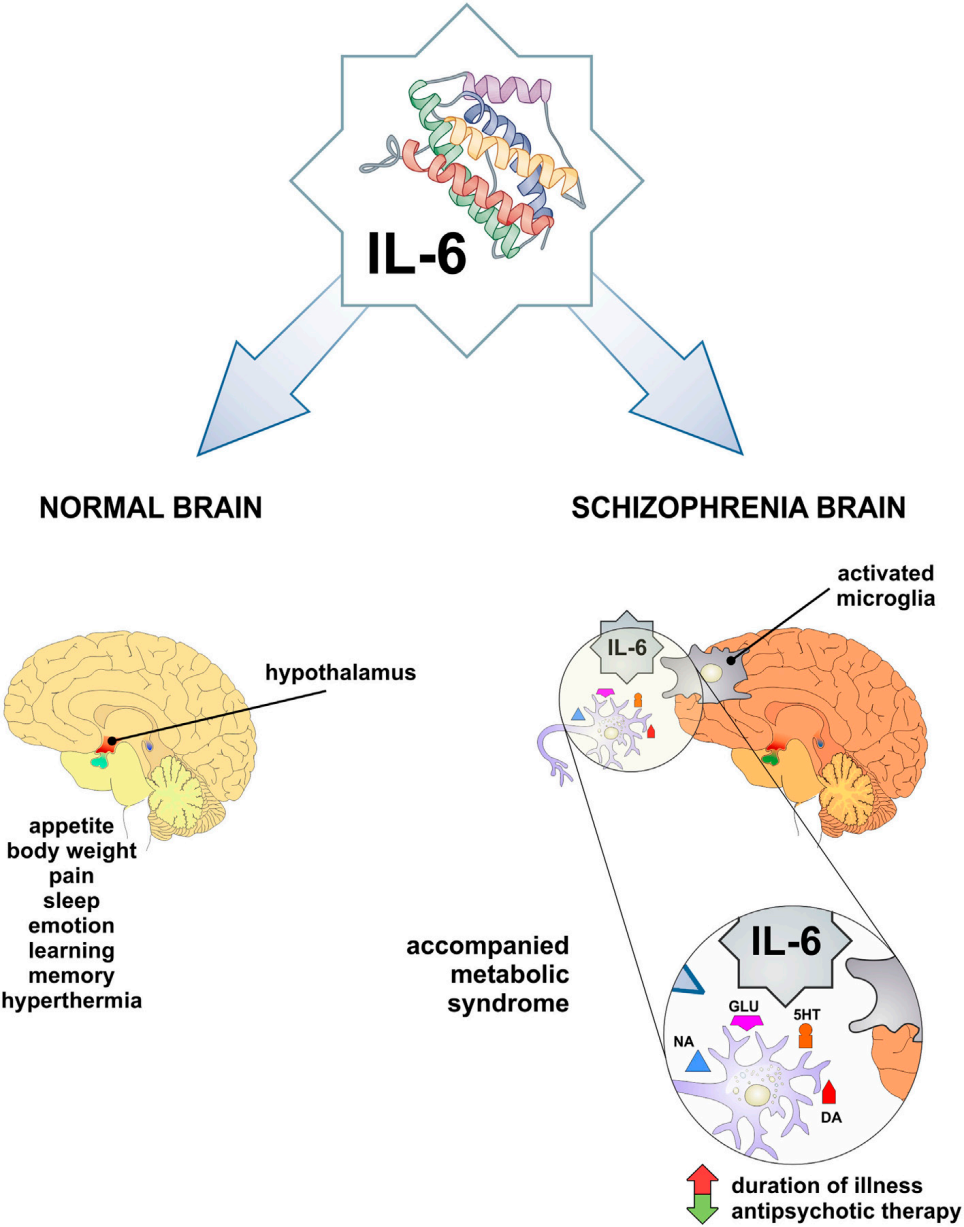
IL-6 as a mediator in physiology and pathology of CNS. Both physiologic functions observed low-grade inflammation-related phenomena and may be involved in obesity and schizophrenia. In pathological conditions, such as increased IL-6 level in CNS and periphery may be involved in schizophrenia and accompanied metabolic syndrome. IL-6, interleukin-6; DA, dopamine; 5HT, serotonin; NA, noradrenaline; GLU, glutamate.

Interleukin-6 contributes in the normal brain functioning (Figure 1): it is involved in the body weight control, food intake, and energy expenditure, it stimulates the pituitary–adrenal axis, has a role in pain, sleep-wake behavior, emotional reactivity, learning, and memory [reviewed in Ref. (35)]. Pyrogenic effects of IL-6 have been widely explored (36) and sickness behavior was observed in association with higher IL-6 levels in the peripheral circulation and the liver of a mouse (37).

Interleukin-6 exerts its effects on neurotransmission of catecholamines, by intensifying dopaminergic and serotonergic turnover in hypocampus and frontal cortex (38, 39). Although, there was no effect of IL-6 on noradrenaline, reversely this neurotransmitter could induce expression of IL-6 in glial cells (40). IL-6 and other pro-inflammatory cytokines activate kinurenine pathway, involved in glutamatergic neurotransmission [reviewed in Ref. (41)].

While IL-6 can have protective properties in many infections, its activity seems to be a key in maintaining the chronic inflammation in model of autoimmune encephalitis and various neurological diseases when IL-6 is overexpressed in the central nervous system [reviewed in Ref. (11)]. Population-based longitudinal studies reported associations of higher serum IL-6 with future risks for depression and psychosis (42). Increased levels of IL-6 were observed in acutely ill patients with schizophrenia, bipolar mania, and major depressive disorder and significantly decreased following treatment in schizophrenia and major depressive disorder, so one may speculate about common stress-related phenomenon across acute phases of these disorders (43).

## Possible Role of IL-6 in Schizophrenia

Interleukin-6 has been widely studied in different aspects of schizophrenia: its onset and progression, association with different clusters of symptoms, response and resistance to the treatment, and metabolical and other comorbid states. IL-6-174G/C polymorphism showed to be associated with increased IL-6 plasma levels and represent a risk factor for schizophrenia (44). IL-6 gene expression in first-episode psychosis is in significant negative correlation with BDNF gene expression and associated with a smaller left hippocampal volume (45). The meta-analysis of Baumeister et al. (46) provide strong evidence that traumatic events have significant impact on the inflammatory immune system. Further, IL-6 is included in potential molecular pathway that leads to development of mental disorders and somatic states later in life. Induced viral or bacterial infection with IL-6 in pregnant mice produces intermediate phenotypes that are related to adult offspring schizophrenia (47). Also, increased levels of IL-6 were found only in those patients with schizophrenia that had a positive childhood trauma history (48). The Avon Longitudinal Study of Parents and Children has recently reported twofold increased risk of psychotic disorder at age 18 years for subjects who had higher IL-6 serum levels at age 9 years, in a dose–response manner (42).

Previous studies have presented conflicting results regarding the levels of IL-6 in schizophrenia. Some authors did not report any alterations in central nervous system (49, 50) and serum (51–55). Elevated levels of IL-6 have been measured in the cerebrospinal fluid of schizophrenia patients by others (56–58). The first meta-analysis of cytokine levels in schizophrenia patients has concluded that IL-6 levels are increased (59), but recent meta-analysis has pointed out that IL-6 is increased in first-episode psychosis and acute relapse, and can be used as a state marker of schizophrenia (60). This has been confirmed by elevated IL-6 level in subjects with at-risk mental state (ARMS) and suggested that it can be used as a marker in prodromal period (61). On the contrary, our findings (62) did not confirm elevation of IL-6 in first-episode psychosis and schizophrenia in relapse. Additionally, Ganguli et al. (63) establish the positive correlation between IL-6 level and illness duration. Therefore, Potvin et al. (59) assumed that the fluctuation of IL-6 level in schizophrenia may be relevant for its pathogenesis. Taking all this into account, it is of great importance to mark the exact period in the evolution of this chronic and deteriorating disorder, in order to understand the possible different roles of IL-6 in acute inflammation, chronic inflammation, and/or autoimmunity in natural history of schizophrenia (Figure 1).

Positive correlation between IL-6 plasma levels and the positive symptoms severity were suggested in subjects with ARMS (61) and war veterans with schizophrenia (64). Levels of IL-6 mRNA from peripheral blood mononuclear cells were found to be elevated in patients with worse positive symptomatology (65). Others presented results of positive correlation between IL-6 serum levels and negative symptoms severity in drug-naive male patients with schizophrenia (66). Recently, it has been shown that individuals with schizophrenia have higher plasma levels of IL-6 that are correlated with depressive symptoms and worse mental and physical well-being (67). Higher IL-6 levels showed to be related with cognitive decline in schizophrenia (44). These neurobiological findings could direct the remodeling of categorical approach (68) and dimensional approach (69) into some new concepts of schizophrenia syndrome.

## Role of IL-6 in the Metabolic Functions

Obesity itself leads to systemic inflammatory response, called metaflammation, originated from metabolic tissues such as adipose tissue, pancreatic islets, liver, muscle, and brain (70). In response to metabolic stress triggered by the excess of nutrients, expanding adipose tissue infiltrates Th1 lymphocytes, NKT cells, and classically activated macrophages that mediate the development of metabolic abnormalities (71–73). Macrophages may be activated in different ways, which favor microbicidal and pro-inflammatory functions (called classically activated macrophage, M1), or in contrast, reparative, and anti-inflammatory functions (called alternative activated macrophage, M2). On the other hand, Treg cells, Th2 lymphocytes, and alternatively activated macrophages exert protective role in nutrient excess-induced inflammation (74, 75). Pro-inflammatory macrophages are the major source of TNF-α, IL-1β, and IL-6 in metabolic tissues that mediate impaired glucose utilization and attenuate insulin sensitivity in both paracrine and endocrine manner (76). Systemic level of IL-6 strongly correlates with obesity and insulin resistance and serum concentrations of IL-6, sIL-6R, and gp130 are elevated in patients with metabolic syndrome (MetS) and related cardiovascular disorders (77). On the contrary, the production of IL-6 by skeletal muscles during exercise is found to be protective (78). Additionally, the deletion of gene encoding IL-6 impairs systemic insulin sensitivity and enhances hepatic inflammation (79). In accordance with these pleiotropic properties of IL-6, it seems that it can exhibit different effects in tissue-specific manner.

Apart from the impact on adipose tissue expansion during obesity, IL-6, as the most important regulator of numerous functions in central nervous system (35), is widely expressed in hypothalamic region that regulates appetite and energy intake (80). The expression of IL-6 in central nervous system negatively correlates with the expansion of adipose tissue during obesity (81). It was previously shown that mice lacking gene encoding IL-6 develop mature onset obesity, suggesting an important role of IL-6 in the regulation of body weight (82, 83). Intracerebroventricular administration of IL-6 increases energy expenditure thus demonstrating central anti-obesity effects of IL-6 (82, 83). The recent data show that IL-6 exhibits anti-inflammatory properties during obesity by promoting IL-4-dependant alternative macrophage polarization thus contributing to attenuation of obesity-induced inflammation and regulation of glucose homeostasis (15).

## IL-6 as a Linkage Between Schizophrenia and Metabolic Syndrome

Metabolic abnormalities including obesity and obesity-related disorders such as impaired glucose tolerance, type 2 diabetes, and cardiovascular disease are strongly associated with psychotic diseases, in particular schizophrenia (84). Patients with schizophrenia are at higher risk to develop MetS, although it is not clear weather this is a disease-inherited state or the side effect of widely used antipsychotic medications (85, 86). Schizophrenia and type 2 diabetes could be associated independently of antipsychotic treatment, possibly based on the common genetic background (87). There are evidences that drug-naive patients in the first episode of schizophrenia have impaired glucose tolerance (88). Moreover, in the first episode of schizophrenia the elevated circulating insulin-related peptides were found, with no difference in glucose levels (89).

However, numerous studies have confirmed that both schizophrenia and MetS underlie chronic low-grade inflammation indicating that disturbances in immune response might be involved in concurrent onset of both conditions (90–92). Increase in adipose tissue activity could contribute to the inflammation seen in schizophrenia. Also, low-grade inflammation independent of adipose tissue activity have been associated with low-physical inactivity, inadequate dietary choices, smoking, and stress, which are often seen in schizophrenia patients (93–95).

Cytokines that are important in glucose utilization and insulin sensitivity appear to be elevated and might be involved in pathogenesis of schizophrenia (59, 60, 96). It has been shown that patients with schizophrenia have higher plasma levels of IL-6 and significant correlation of cytokine plasma levels with body mass index was established (67).

These metabolic abnormalities can correlate with both schizophrenia and antipsychotic treatment, possibly based on the alterations of systemic levels of different cytokines and adipokines (96). Antipsychotics can increase rates of obesity, with consequent upregulation of IL-6, and leptin (97). In rodent and human studies, there are evidence for an association between leptin, cognition, and behavior. Leptin modulates activity of mesolimbic dopaminergic neurons in the hypothalamus, which is especially important in schizophrenia (98). Trujillo et al. (99) showed that leptin production was increased by IL-6 in human adipocyte cultures, but others observed IL-6 inhibitory function or no effect on leptin production (100, 101). It appears that leptin has neuroprotective role, but in antipsychotic-induced leptin resistance and in obesity these neuroprotective properties are not so obvious (102, 103). Therefore, cytokine changes that are associated with antipsychotic treatment could be a consequence of weight gain (104). We did not find significant difference in the serum level of IL-6 in psychotic patients compared with healthy control (62). However, we observed that serum level of IL-6 had significantly decreased after antipsychotic treatment in patients with first-episode psychosis and schizophrenia in relapse (105). These data suggest that decreased levels of IL-6 following antipsychotic therapy could be predisposing factor for the development of obesity and obesity-related metabolic disorders in schizophrenia (Figure 1).

Novel insight into pathogenesis of psychotic disorders indicates that gut microbiota could have a role in cognitive and behavioral patterns and affects the development of MetS through not entirely known mechanisms (106, 107). Commensal microorganisms trigger activation of innate immune cells such as dendritic cells and macrophages in lamina propria, following increased production of pro-inflammatory IL-1β, IL-6, IL-23, and possibly IL-12, thus contributing to the polarization of adaptive immune response toward Th17 or Th1 type, respectively (108). Increased intestinal inflammation was observed in patients with schizophrenia, more significantly before the initial administration of antipsychotics (109). Gut microbial composition affects systemic cytokine concentrations and possibly, by this gut-brain communication, alters the behavior in schizophrenia [reviewed by Khandaker et al. (110)].

## IL-6 as a Potential Therapeutic Target in Schizophrenia

Clinicians noticed altered immune response in patients with schizophrenia long before antipsychotics’ era [reviewed in Ref. (111)]. This finding indicated that antipsychotics would affect not only the schizophrenia outcome, but additionally would modify the immunity of treated patients. All available antipsychotics treat the symptoms of schizophrenia by blocking D2 receptors, but also regulate the serotonin and glutamate neurotransmission. Efficacy and side effects of antipsychotics cannot be completely explained by neurotransmission theory and it is well known that they exhibit neurotrophic, neurogenetic, and neuroprotective properties (112–114). Several studies reported that antipsychotics decrease systemic values of pro-inflammatory cytokines (114–116). Some researchers found an increase of anti-inflammatory cytokine IL-10 in sera of patients treated with antipsychotics (117) and we showed that increased levels of TGF-β stay elevated after antipsychotic therapy in first-episode psychosis and schizophrenia in relapse (62, 105). Taken together, it appears that antipsychotics have additional anti-inflammatory properties.

It was previously suggested that treatment resistance in schizophrenia is associated with IL-6 elevated levels (118). Several cytokines, including IL-6, can predict a treatment response in first-episode psychosis (119). Decrease of systemic value of IL-6, together with favorable clinical outcome following anti-psychotic therapy, is the dominant phenomenon in most studies (59, 116, 120–123). Researchers found a significant positive correlation between the concentration of IL-6 in sera and psychopathology at the onset, as well as after the administration of antipsychotics (60). In the post-mortem orbitofrontal brain studies in people with schizophrenia, IL-6 mRNA significantly positively correlated with antipsychotic lifetime and daily mean intake (124). Few studies revealed that clozapine affects the increase of IL-6 in the plasma during the 2 weeks, but not the longer treatment (125–128), while other, comprehensive studies showed that atypical antipsychotic risperidone or the typical antipsychotic haloperidol do not significantly affect serum levels of IL-6 in patients with schizophrenia (117, 129). Further, decrease of IL-6 in the plasma of patients with exacerbation of schizophrenia was shown after discontinuation of the haloperidol therapy (130). The peripheral low-grade inflammation was observed in animal model after olanzapine treatment, correlated with upregulation of IL-6 in hypothalamus and adipose tissue (white and brown), and enhanced average size of adipocyte and macrophage infiltration level (131).

Clinical studies pointed out the beneficial effects of immunomodulatory therapy in schizophrenia, especially in early stage of the disorder (132) with respect to symptoms severity (133, 134), and in improving cognitive impairment in patients with schizophrenia (135). Anti-IL-6 drugs have been developed and already used for treatment of various diseases and cancers, such as CNTO328 chimeric anti-IL-6 monoclonal antibody (mAb) (siltuximab) and anti-IL-6R mAb, atlizumab (also called tocilizumab) (136). Ingested tocilizumab can inhibit experimental autoimmune encephalitis by decreasing pro-inflammatory Th1 cytokines and increasing Th2 anti-inflammatory cytokines (137). In accordance with important role of IL-6 in regulation of metabolic homeostasis, this kind of therapy might have side effects, such as significant weight gain followed by hypertrygliceridemia and hypercholesterolemia in patients treated with IL-6R neutralizing antibody tocilizumab (138). Blocking of IL-6 trans-signaling, while classical IL-6R signaling stays intact is important for the maintenance of gut mucosal integrity and epithelial regeneration [reviewed by Hunter and Jones (11)].

One of the possibilities in drug development for the treatment of schizophrenia might be the tissue-specific IL-6 blockade, thus avoiding systemic side effects of this kind of treatment. IL-6 plays significant role in disease genesis and progression, and the use of specific inhibitors may not only be beneficial for exacerbation and alleviation of positive symptoms, but in particular to possible attenuation of cognitive impairment in patients with schizophrenia.

## Conclusion

Interleukin-6 orchestrates the innate and acquired immunity, but all these effects are context dependent and tissue-specific role of IL-6 in central nervous system and other metabolite tissues must be considered. The functional dichotomy of IL-6 may play a critical role in maintaining the balance between pro- and anti-inflammatory responses. It seems that IL-6 can have a phase specific role in schizophrenia evolution, in the context of acute inflammation, chronic inflammation, and/or autoimmunity. Limitation is that there is between-study heterogeneity and most valuable studies are those comparing different phases of illness and considering influence of age, sex, illness duration, BMI, fasting status, and applied therapy. Now it seems to be clear that metabolic dysregulation in terms of glucose metabolism alteration (139) or lipide profile disturbance (140) occurs already in antipsychotic-naïve patients with first-episode psychosis. IL-6 and leptin activity in hypothalamus could explain co-occurrence of schizophrenia and metabolic syndrome. Current research data about the role of microbiome in schizophrenia is still modest, but antipsychotic-induced alterations of the gut microbiota and metabolic changes should also be thoroughly explored (141). Treatment-resistant schizophrenia is associated with increased IL-6 sera level, and the relationship between higher IL-6 level and cognitive decline in schizophrenia has been observed, thus implicating the impact of IL-6 on behavioral aspects of schizophrenia. Beneficial effects of immunomodulatory therapy in schizophrenia have been already shown and the use of tissue-specific inhibitors of IL-6 or other IL-6-targeted therapy could possibly be useful in the treatment of schizophrenia and comorbid somatic states.

## Author Contributions

All authors were included in the designing of the manuscript, drafting the work, critical revision, and final approval for all aspects of the work and the final version to be published.

## Conflict of Interest Statement

The authors declare that the research was conducted in the absence of any commercial or financial relationships that could be construed as a potential conflict of interest. The reviewer KK and handling editor declared their shared affiliation.
